# Nutrient Enrichment and Food Web Composition Affect Ecosystem Metabolism in an Experimental Seagrass Habitat

**DOI:** 10.1371/journal.pone.0007473

**Published:** 2009-10-15

**Authors:** Amanda C. Spivak, Elizabeth A. Canuel, J. Emmett Duffy, J. Paul Richardson

**Affiliations:** Virginia Institute of Marine Science, College of William and Mary, Gloucester Point, Virginia, United States of America; University of North Carolina at Chapel Hill, United States of America

## Abstract

**Background:**

Food web composition and resource levels can influence ecosystem properties such as productivity and elemental cycles. In particular, herbivores occupy a central place in food webs as the species richness and composition of this trophic level may simultaneously influence the transmission of resource and predator effects to higher and lower trophic levels, respectively. Yet, these interactions are poorly understood.

**Methodology/Principal Findings:**

Using an experimental seagrass mesocosm system, we factorially manipulated water column nutrient concentrations, food chain length, and diversity of crustacean grazers to address two questions: (1) Does food web composition modulate the effects of nutrient enrichment on plant and grazer biomasses and stoichiometry? (2) Do ecosystem fluxes of dissolved oxygen and nutrients more closely reflect above-ground biomass and community structure or sediment processes? Nutrient enrichment and grazer presence generally had strong effects on biomass accumulation, stoichiometry, and ecosystem fluxes, whereas predator effects were weaker or absent. Nutrient enrichment had little effect on producer biomass or net ecosystem production but strongly increased seagrass nutrient content, ecosystem flux rates, and grazer secondary production, suggesting that enhanced production was efficiently transferred from producers to herbivores. Gross ecosystem production (oxygen evolution) correlated positively with above-ground plant biomass, whereas inorganic nutrient fluxes were unrelated to plant or grazer biomasses, suggesting dominance by sediment microbial processes. Finally, grazer richness significantly stabilized ecosystem processes, as predators decreased ecosystem production and respiration only in the zero- and one- species grazer treatments.

**Conclusions/Significance:**

Overall, our results indicate that consumer presence and species composition strongly influence ecosystem responses to nutrient enrichment, and that increasing herbivore diversity can stabilize ecosystem flux rates in the face of perturbations.

## Introduction

Theory predicts that interactions between resource availability and trophic structure will influence biomass distribution across trophic levels and ecosystem functioning [1]–[5]. In coastal waters, nutrient enrichment often results in increased algal biomass, reduced water clarity, and loss of submerged macrophytes [6]–[8]. These effects can be attenuated or exacerbated by consumers depending on the number of trophic links and the consequent presence and strength of a trophic cascade [3], [9]–[11]. By extension, shifts in resource levels or trophic structure that influence consumer and plant abundances and nutritional quality may also alter nutrient dynamics and ecosystem productivity [3], [12]–[15]. The relative importance of resource availability (i.e. bottom-up controls) and trophic structure (i.e. top-down controls) in determining community and ecosystem properties has long been a subject of keen interest in freshwater [9], [16], [17], terrestrial [18]–[21], and marine [22], [23] ecosystems. More recently, biodiversity has been suggested to stabilize ecosystem responses to perturbations at the top [3] and bottom [2], [5] of the food web. To explore this idea, we used experimental seagrass ecosystems to test whether grazer diversity can modify the effects of nutrient enrichment and predation on primary producer abundance, ecological stoichiometry, and ecosystem metabolism.

Herbivores occupy a key node in many food webs, serving as the link from primary producers to higher trophic levels as well as potential regulators of plant biomass and community composition. In seagrass food webs, for example, grazing invertebrates are both influenced by changes in resource levels and trophic structure and, in turn, influence how those changes are propagated through the food web. Increased nutrient supply can stimulate growth rates and increase the quality of algae, enhancing secondary production of grazers [24]–[26]. In turn, grazers contribute to nutrient cycling by storing nutrients as biomass and excreting elements unused for growth and metabolism [13], [27]–[29]. Conversely, changes in top-down pressure can alter community composition by changing prey abundance, behavior, and stoichiometry [30]–[32]. When predators are absent, algal biomass is often reduced and grazer-mediated nutrient recycling is increased. Whether such changes in resource levels and/or trophic structure are propagated through the food web can depend on grazer diversity and food preferences [3], [14], [33]–[35]. Thus, the outcome of changes in resource availability or predator abundance on biomass and elemental cycling can depend on herbivore community composition [1], [3].

In soil and sedimentary environments ecosystem-level effects of food web structure and resources are complicated by interactions with the below-ground microbial community [15], [36]–[38]. The stoichiometry of organic matter (OM) produced above-ground can strongly influence benthic community structure as well as sediment nutrient dynamics. In particular, deposition and incorporation of algal detritus into the sediments increases sediment organic matter (SOM) quality and lability, which are partial determinants of sediment microbial activity. Bacteria return a portion of remineralized nutrients to the water column while still retaining some nutrients to maintain optimum elemental balance [39]. High rates of OM deposition may also stimulate microbial activity leading to sediment anoxia and, consequently, nitrate uptake (denitrification) and carbon burial [40], [41]. High rates of OM deposition may also affect the availability of phosphate, which is chemically bound and biologically unavailable in oxic sediments but is desorbed as anoxic conditions develop [42]. Thus, depending on the quantity and quality of OM delivered to the benthos, sediments may be a source or a sink of inorganic nutrients.

Previous experiments in seagrass systems demonstrated that resource availability and food chain length can influence above-ground biomass distribution between trophic levels [43]–[47], SOM composition and quality [48], [49], and gross ecosystem productivity [50]. But it remains unclear whether responses to perturbations at the top and bottom of the food web are modified by community composition and diversity. To assess the effects of resource availability and food web composition on seagrass ecosystem structure and functioning, we factorially manipulated water column nutrient concentrations, food chain length, and herbivore species diversity and measured their effects on above-ground biomass, plant and grazer stoichiometric ratios, and ecosystem metabolism. We predicted that nutrient enrichment would increase primary producer biomass, gross ecosystem production (GEP), and the quality of OM deposited to the sediments, thereby increasing sediment microbial activity, leading to higher fluxes of dissolved inorganic nitrogen (DIN). Secondly, we expected that grazers would reduce algal abundance and recycle the consumed nutrients back into the water column as DIN. Finally, we predicted that increasing grazer diversity would dampen the trophic cascade previously demonstrated in this system [34], [48], [50], and therefore stabilize primary producer abundance, GEP, SOM quality, and sediment DIN flux.

## Materials and Methods

### Experimental Design

We conducted a mesocosm experiment to determine the main and interactive effects of nutrient enrichment, grazer presence and species richness, and food chain length (presence vs. absence of a predator) on the accumulation of primary producer and grazer biomass, flux rates of dissolved oxygen and inorganic nutrients, and the elemental ratios of seagrass, algae, and invertebrate grazers. Water column nutrient levels were manipulated by adding Osmocote™ (N∶P∶K 3∶1∶2) slow release fertilizer to half of the tanks. Grazer species diversity varied across four levels (0, 1, 3, or 5 species). The highest grazer diversity level contained five amphipod species present in the York River, VA, at the time of the experiment, each replicate of the intermediate level contained a random combination of three species, and the lowest diversity level only had the most abundant species, *Gammarus mucronatus*. The remaining four grazer taxa were: *Elasmopus levis*, *Melita nitida*, *Ampithoe valida*, and *Sympleustes* spp. Thus the grazer diversity gradient simulated loss of rare species and increasing dominance by the most abundant species. Food chain length was manipulated by exposing parallel sets of grazer treatments to a generalist predator, the blue crab, *Callinectes sapidus*. The 16 treatments were replicated 3 times each for a total of 48 mesocosm tanks.

We conducted this experiment in a mesocosm system because of the severe challenges associated with manipulating mesograzer species composition and performing the *in situ* ecosystem flux measurements in a natural eelgrass bed. Although mesocosm systems have limitations (e.g. [51], [52]), our experimental infrastructure simulated well several aspects of biotic and abiotic conditions in the field [44]. For instance, water temperature averaged 23°C at the Goodwin Island eelgrass bed (Moore unpubl. data) versus 25°C in the mesocosm system. Additionally, similar responses of primary producer biomass and surface sediment characteristics between previous field [49] and mesocosm [48], [50] experiments suggest that the conditions in the mesocosms reflect the natural environment in important ways.

The outdoor mesocosm experiment was conducted over five weeks during summer 2006 in 120-liter translucent fiberglass tanks that were continuously supplied with water from the York River estuary. Water passed through a sand filter and then through 150 µm mesh before filling ‘dump buckets’ which regularly spilled into the tanks, providing turbulence and aeration. The filtering process eliminated larger animals and debris and minimized invasion by non-target animals while permitting passage of invertebrate larvae and algal spores, which often colonized the tanks. The tanks were filled with a sand – mud mixture (9∶2), initially averaging 0.80% (±0.18 SE) OM content, to a depth of 10 cm. In contrast with previous experiments [48], [50], we chose to use a sediment substratum with approximately 1% OM to facilitate *Zostera marina* transplant success and growth [53]. In each mesocosm, 100 pre-weighed eelgrass (*Z. marina*) shoots were planted after being rinsed clean of grazers and epiphytes with fresh water. In addition we planted artificial seagrass units, consisting of a green ribbon tied to vexar mesh, as a standardized substratum for accumulating epiphytic growth.

Sixteen days after planting, grazing invertebrates were added to each grazer mesocosm. The five-species treatment received 18 individuals of each species, the three-species treatment had 30 individuals of each species, and the one-species treatment had 90 individuals of *G. mucronatus*, following a replacement series design. We chose this gradient in herbivore diversity for two reasons. First, a goal of our experiment was to understand how ecosystem processes change following a realistic sequence of species loss and subsequent community reorganization. Second, we manipulated initial densities but allowed the grazers populations to grow and adjust naturally to the presence or absence of other grazer species. The initial density of grazers probably did not determine final grazer densities since the grazers reproduce at high rates and likely reached carrying capacity in the mesocosm tanks by the end of the five week experiment [54]. For instance, a previous manipulation in this system using a combined replacement-additive design demonstrated that initial differences in grazer densities had little effect as grazer biomass converged across all density and diversity treatments after four weeks [54].

Eleven days after grazer additions, two juvenile blue crabs (20–40 mm carapace width) were added to each predator treatment. Each nutrient treatment received 200 g of fertilizer in the first two weeks and 100 g every week thereafter. Preliminary trials revealed that dissolved inorganic nitrogen (DIN) concentrations peaked within 24 h after fertilizer addition and then declined to a constant level for four days before falling again. Fertilizer additions were refreshed twice weekly to maintain elevated and relatively constant nutrient levels. Fertilizer was dispensed through two perforated PVC tubes suspended in the tanks. Water column nutrient concentrations were monitored each week by measuring NH_4_^+^ concentrations from five randomly chosen tanks of each nutrient treatment.

The five-week experimental incubation time minimized the risk of invasion by non-target animals, prevented the complete consumption of eelgrass by the grazers, and permitted major changes in animal (one to two grazer generations) and plant community development and in surface sediment characteristics [45], [48], [50]. After five weeks, we measured whole ecosystem fluxes of dissolved oxygen (DO), NH_4_^+^, NO_x_, and PO_4_^−3^, as well as primary producer biomass and the carbon and nitrogen ratios of sediments, primary producers, and invertebrate grazers (see below).

### Biomass sampling

To determine primary producer biomass at the end of the experiment we collected above-ground seagrass blades, macroalgae, artificial seagrass blades (to estimate epiphytic chlorophyll *a* (chl *a*) accumulation), and sediments for benthic chl *a*. Because the tanks were a flow-through system, we did not measure phytoplankton abundance. Seagrass and algae were frozen (−20°C) until analysis at which point they were dried (60°C) and combusted (400°C) to determine ash-free dry mass (AFDM). Epiphytic chl *a* was extracted from the artificial seagrass blades in a 90∶10 (v∶v) acetone ∶ methanol solution for 24 h at −20°C; samples were processed according to Douglass et al. [46]. For benthic chl *a*, three sediment cores (1.5 cm diameter) were collected from each tank and the upper 1 cm was removed. The three sub-samples were combined in a pre-combusted (450°C) scintillation vial, frozen (−20°C), and analyzed within six weeks of collection [55]. Since benthic microalgal distribution can be patchy, we used composite samples to increase the likelihood that the benthic chl *a* concentrations represented the entire surface sediment in each mesocosm tank.

Invertebrate grazers were collected at the end of the experiment and stored in ethanol. Sub-samples were analyzed for grazer species identity, abundance, and size class. Grazer ash-free dry mass (AFDM) was determined using previously established empirical relationships between body mass and size of sieve on which the animal was retained [56].

### Ecosystem metabolism

We measured fluxes of dissolved oxygen (DO), NH_4_^+^, NO_x_, and PO_4_^−3^ to characterize whole-ecosystem metabolism. Four days before the end of the experiment, we sampled approximately every hour over two four-hour incubation periods, one during the day (10:00–14:00 h) and another at night (22:00–02:00 h). Immediately prior to the incubation period, the water supply was shut off and clear plastic sheeting (2 mm thickness) was placed on the water's surface to minimize oxygen exchange with the atmosphere. Before each measurement the water was stirred to disrupt any stratification. DO concentrations were measured using a YSI datasonde. Water samples (25 mL) for NH_4_^+^, NO_x_, and PO_4_^−3^ concentrations were filtered through a pre-combusted (450°C) glass fiber filter and frozen (−20°C) until analysis by standard methods using a Lachat auto-analyzer [57]–[59]. We calculated the slope of change in concentration versus the time elapsed and divided this by the area of the tank to obtain flux. Hourly day and night rates were scaled to the volume of the mesocosm tanks (120 l) and to 14 h of light and 10 h of darkness to estimate daily summertime gross and net ecosystem production of DO and daily net flux rates of inorganic nitrogen and phosphorus. DIN concentrations were calculated by summing NH_4_^+^ and NO_x_. To calculate respiration, hourly nighttime oxygen consumption was scaled to 24 h; respiration and net ecosystem production were converted to carbon units using an assumed respiratory coefficient (RQ) of 1.0 [60], [61]. The ratio of production to respiration (P∶R) was calculated by dividing estimated GEP by respiration. We are confident that our flux rates reflect biological processes within the experimental tanks because the measured flux rates were much higher than the dissolution rate of Osmocote™ (0.24 µM NH_4_^+^ h^−1^).

### Elemental composition of primary producers, grazers, and sediments

After five weeks, we collected samples of seagrass blades, macroalgae, grazers, and sediments from each mesocosm to assess the effects of water column nutrient enrichment on elemental composition of biomass. Twenty individuals of each of the amphipods *G. mucronatus* and *A. valida* were collected from the treatments in which they were originally stocked. The top 1 cm of three sediment cores (2.6 cm diameter) was collected from each tank and combined to form a composite sample of surface sediment. All samples were placed in separate pre-combusted (450°C) vials and stored at −20°C until analysis for total organic carbon (TOC) and total nitrogen (TN) content by standard methods with a Fisons Flash Elemental Analyzer (Model 1112) after removing inorganic carbon [62]; acetanilide was the standard. Molar elemental ratios were calculated by first normalizing TOC and TN to the molar weight of carbon and nitrogen, respectively, and then dividing molar TOC by molar TN. We did not measure the phosphorus content of the grazers or sediments.

### Statistical analyses

Effects of experimental treatments on each response variable were analyzed using fully factorial three-way analysis of variance (ANOVA, SAS version 9.1 for Windows), with grazer treatment (df = 3), food chain length (i.e. predator presence or absence, df = 1) and nutrient level (df = 1) as fixed factors. Data were logarithmically transformed as necessary to maintain homogeneity of variance as determined by the Cochran's C test. From the ANOVAs, we calculated the magnitude of main and interactive effects (ω^2^, estimated proportion of variance explained by the experimental variable [63]). One sample was excluded from analyses of *Z. marina* and macroalgal biomass (nutrient, no-crab, five-grazers) while four samples were excluded from analysis of grazer biomass (no-nutrients, no-crabs, five-grazers; nutrients, no-crabs, five-grazers; nutrients, crabs, one-grazer; nutrients, crabs, three-grazers) due to sample loss. The type III sum of squares (SS) results from the ANOVA model are reported.

Our analyses produced a substantial number of separate statistical tests, which could increase the risk of spurious correlations. In considering how to minimize this risk, we carefully considered, and ultimately rejected, the Bonferroni and related procedures due to their several weaknesses, including a higher probability of type II statistical errors and the subjectivity of deciding what constitutes an appropriate level at which to aggregate the tests [64], [65]. However, since the possibility of type I errors remains for any individual test, we strive to focus on broad patterns rather than on the details of individual comparisons. As such we de-emphasize individual p values and instead compare the relative importance of the manipulated variables (nutrients vs. grazers vs. crabs) using the estimated magnitude of effect, ω^2^. In the discussion section, we highlight ecologically important results and downplay statistically significant, yet ecologically negligible results.

To help interpret the drivers of ecosystem flux rates, we performed multiple linear regressions of daily GEP, respiration, and fluxes of DIN and PO_4_^−3^ against the abundances of the major primary producers. To detect correlations between the flux rates, we performed a multiple linear regression of GEP against DIN and PO_4_^−3^. Simple linear regressions of GEP, respiration, and flux rates of DIN and PO_4_^−3^ against sediment C∶N were also performed. In addition, we regressed respiration against net ecosystem production and sediment C∶N to understand whether respiration was related to autochthonous OM production or bulk SOM quality.

## Results

### Nutrient concentrations

During the first two weeks of the experiment 200 g of fertilizer were added to each nutrient treatment, resulting in an average NH_4_^+^concentration of 29.23 µM (±5.45 SE). For the remaining three weeks each nutrient treatment received 100 g of fertilizer and the average NH_4_^+^concentration fell to 14.37 µM (±1.32 SE). Concentrations of NH_4_^+^ were 0.95 µM (±0.25 SE) and 2.58 µM (±0.57 SE) in the unenriched treatments during weeks 1–2 and 3–5, respectively. Thus, the NH_4_^+^ concentration of nutrient treatments was approximately 30 times ambient during the first two weeks and 5 times ambient during the remaining three weeks. The NH_4_^+^ concentrations in the no-nutrient treatments were typical of late spring and summer conditions in the York River estuary while the concentrations in the nutrient-enriched treatments were similar to or higher than late fall conditions (K. Moore *unpubl. data*).

### Primary producer and herbivore biomass

**Figure 1:**
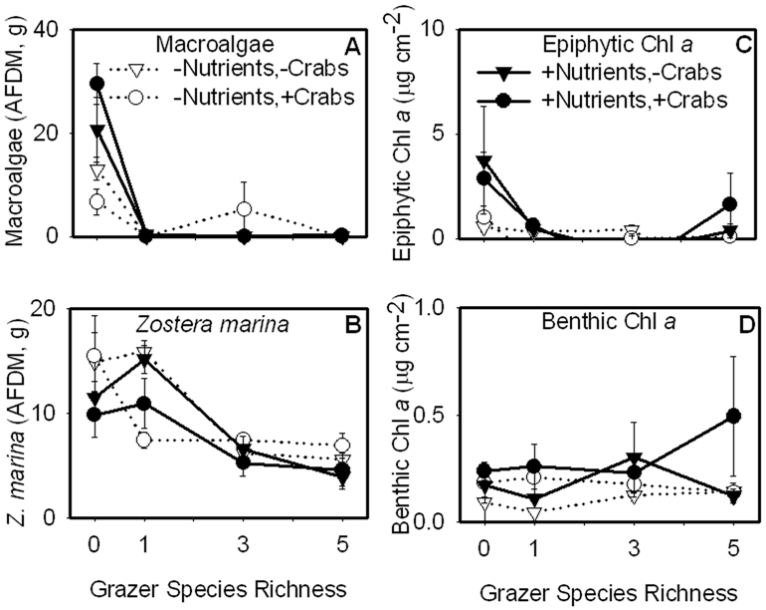
Effects of nutrient enrichment, crab presence, and grazer richness on primary producer biomass. Nutrient enrichment increased macroalgae (A) and epiphytic Chl *a* (C). Grazers reduced abundances of macroalgae (A), *Z. marina* (B), and epiphytic Chl *a* (C). Benthic Chl *a* (D) was unaffected by the experimental manipulations. For this and the following figures, all error bars are standard error and the statistical results are reported in Table 1.

Primary producer biomass was generally reduced by grazers and increased by nutrient additions. Relative to the grazer-free controls, grazers reduced epiphytic microalgae (chl *a*) and nearly eliminated macroalgae (Figs. 1A, C; Table 1). Grazers decreased *Z. marina* biomass in the three and five species treatments, but not in the one grazer species treatment (Fig. 1B). Thus, *G. mucronatus*, the species in the single grazer treatment, was likely not responsible for eelgrass loss in the diverse treatments. The higher grazing impact in diverse grazer assemblages also stabilized *Z. marina* biomass against perturbations, as evidenced by the substantially smaller variation in eelgrass biomass among crab and nutrient treatments with 5 grazer species compared with 1 species (Fig. 1B); this stabilizing effect is supported by the significant interaction between grazer and predator treatments (Table 1). Nutrient additions increased epiphytic chl *a* and macroalgae primarily in the absence of grazers, resulting in a nutrient by grazer interaction. Overall, grazers were considerably stronger determinants of plant and algal biomass (ω^2^ values to 0.62) than predators (ω^2^ to 0.05) or nutrient enrichment (ω^2^ to 0.14), as indicated by the estimated magnitudes of effect (Table 1). Benthic chl *a* was insensitive to food chain length, grazer richness, or nutrient enrichment (Fig. 1D).

**Table 1:** Tests of significance and estimated magnitudes of effects (ω^2^) of nutrient enrichment, food chain length, and grazer species richness and their interactive effects on biomass, elemental ratios, and daily flux rates.

Response	Nutrient	enrichment		Food	chain	length	Grazer	community		Interactions	Model	error
	*p*	MS	ω^2^	*p*	MS	ω^2^	*p*	MS	ω^2^		MS	ω^2^
**Plant Biomass**												
*Z. marina*	0.106	25.79	0.02	0.105	25.87	0.02	**<0.001**	182.20	0.49	PxG 0.028 (0.06)	9.30	0.41
Macroalgae	**0.015**	120.38	0.02	0.458	10.24	0.00	**<0.001**	857.12	0.62	NxG<0.001 (0.13)	18.11	0.21
										NxPxG 0.036 (0.03)		
Epiphytic chl *a*	**0.002**	56.36	0.14	0.267	6.63	0.00	**0.004**	27.73	0.19		5.19	0.69
log Benthic chl *a*	0.213	0.29	0.01	0.080	0.59	0.05	0.767	0.07	0.00		0.18	1.12
**Grazer Biomass**												
log Total grazers	0.189	0.54	0.00	0.371	0.25	0.00	**<0.001**	25.16	0.85		0.30	0.14
*G. mucronatus*	**<0.001**	3.49E+8	0.18	0.057	4.86E+7	0.02	**<0.001**	2.96E+8	0.45	NxG 0.002 (0.10)	1.23E+7	0.27
log Minor grazers	0.901	0.00	0.00	**0.002**	4.18	0.47	0.372	0.22	0.00		0.26	0.92
**Stoichiometry**												
*Z. marina* %TN	**<0.001**	13.32	0.64	0.232	0.15	0.00	**0.033**	0.33	0.03	NxP 0.041 (0.02)	0.10	0.23
										NxG 0.008 (0.05)		
*Z. marina* %TOC	0.405	6.65	0.00	0.843	0.37	0.00	**0.012**	40.16	0.15	NxP 0.038 (0.05)	9.32	0.71
										NxG 0.011 (0.15)		
*Z. marina* C∶N	**<0.001**	1476.16	0.71	0.088	37.54	0.01	0.399	12.27	0.00		12.10	0.28
SOM %TN	0.852	0.00	0.00	0.144	0.00	0.02	**0.034**	0.00	0.12		0.00	0.86
SOM %TOC	0.918	0.00	0.00	**0.031**	0.15	0.07	**0.037**	0.10	0.12		0.03	0.87
SOM C∶N	0.055	4.64	0.04	**<0.001**	20.34	0.25	0.427	1.11	0.00		1.16	0.72
*G. mucronatus* %TN	**0.035**	13.04	0.10	0.662	0.51	0.00	0.256	3.78	0.00		2.62	1.05
*G. mucronatus* %TOC	**0.025**	187.99	0.12	0.855	1.12	0.00	0.136	71.31	0.03		32.89	0.99
*G. mucronatus* C∶N	0.155	2.11	0.03	0.769	0.09	0.00	0.084	2.71	0.07		0.98	1.05
*A. valida* %TN	0.421	3.93	0.00	0.766	0.53	0.00	0.958	0.02	0.00	NxPxG 0.045 (0.07)	5.76	1.37
log *A. valida* %TOC	**0.027**	0.01	0.12	**0.002**	0.02	0.32	0.092	0.00	0.01		0.00	0.65
*A. valida* C∶N	0.299	0.06	0.01	0.905	0.00	0.00	0.477	0.03	0.00		0.05	1.46
**Daily flux rates**												
GEP	**<0.001**	1.03E+5	0.11	0.085	1.76E+4	0.01	**<0.001**	1.59E+5	0.53	PxG 0.027 (0.05)	5570.33	0.31
Respiration	**<0.001**	6.30E+4	0.34	**0.014**	1.04E+4	0.05	**0.001**	1.01E+4	0.14	PxG 0.012 (0.08)	1542.94	0.42
P ∶ R	0.155	0.19	0.01	0.285	0.11	0.00	**<0.001**	1.05	0.38		0.09	0.58
DIN	**<0.001**	5.09E+4	0.63	0.906	6.75	0.00	0.201	779.95	0.01	NxPxG 0.041 (0.04)	476.93	0.29
PO_4_^−3^	**<0.001**	114.04	0.52	0.520	0.87	0.00	0.054	5.85	0.05		2.06	0.46
DIN ∶ PO_4_^−3^	**0.002**	4218.69	0.19	0.403	263.41	0.00	0.506	291.47	0.00		366.53	0.88

For interactions, P refers to crab predators, G to grazers, and N to nutrients; ω^2^ is listed in parentheses. P∶R is the ratio of production to respiration. Significant p values are in bold. *Z. marina* and macroalgal biomass were analyzed as AFDM, g; epiphytic and benthic chl *a* as µg cm^−2^; grazer biomass as AFDM, mg; GEP as mmol O_2_ m^−2^ d^−1^; respiration as mmol C m^−2^ d^−1^; DIN and PO_4_^−3^ as mmol m^−2^ d^−1^. When an interaction was significant, the dataset was divided according to the interaction (i.e. crab predators vs. no predators and nutrients vs. no nutrients) and single factor ANOVAs were run; the results for those tests are in Table S1.

**Figure 2:**
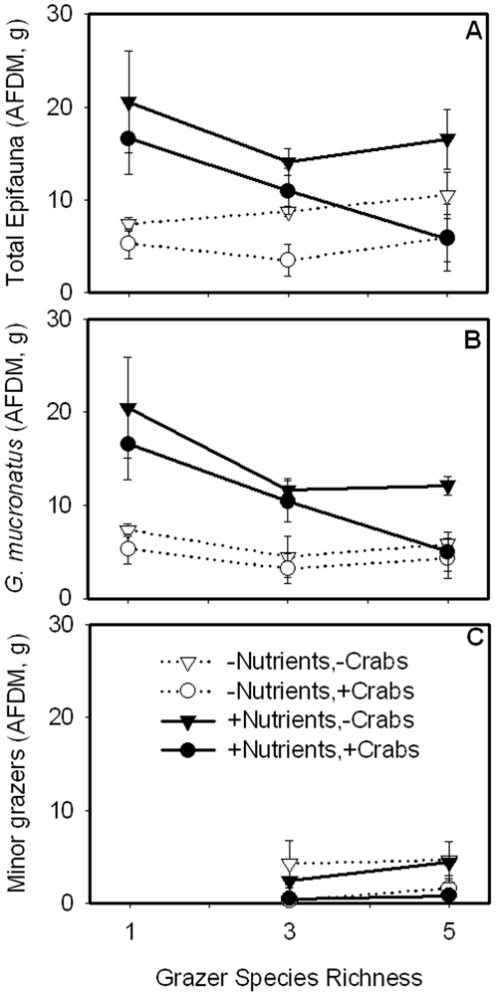
The effects of nutrient enrichment and crab presence on grazer biomass. Total epifaunal biomass (A) was divided into two categories: *G. mucronatus*-only and ‘minor grazers’. Nutrient enrichment increased the biomass of *G. mucronatus* (B) while crab predators reduced the abundance of minor grazers (C).

In the treatments with multiple grazer species, *G. mucronatus* was the most abundant species and the largest contributor to total grazer biomass in both the presence (75–85% of total grazer biomass) and absence (66–68%) of predators (Fig. 2; Table 1). Because *G. mucronatus* was so abundant we divided grazer responses into two categories: *G. mucronatus* only and “minor grazers” (i.e. grazers other than *G. mucronatus*). Nutrient enrichment strongly increased accumulation of *G. mucronatus* biomass (a proxy for secondary production; ω^2^ = 0.18); this effect was strongest in the one-species grazer treatment and resulted in a significant interaction between nutrients and grazer richness. There was no effect of nutrient enrichment on minor grazer biomass. Predators reduced minor grazer biomass (ω^2^ = 0.47) but had little effect on *G. mucronatus*, suggesting that this amphipod was less susceptible than other grazers to predation by blue crabs, as seen previously [34].

### Elemental ratios

**Figure 3:**
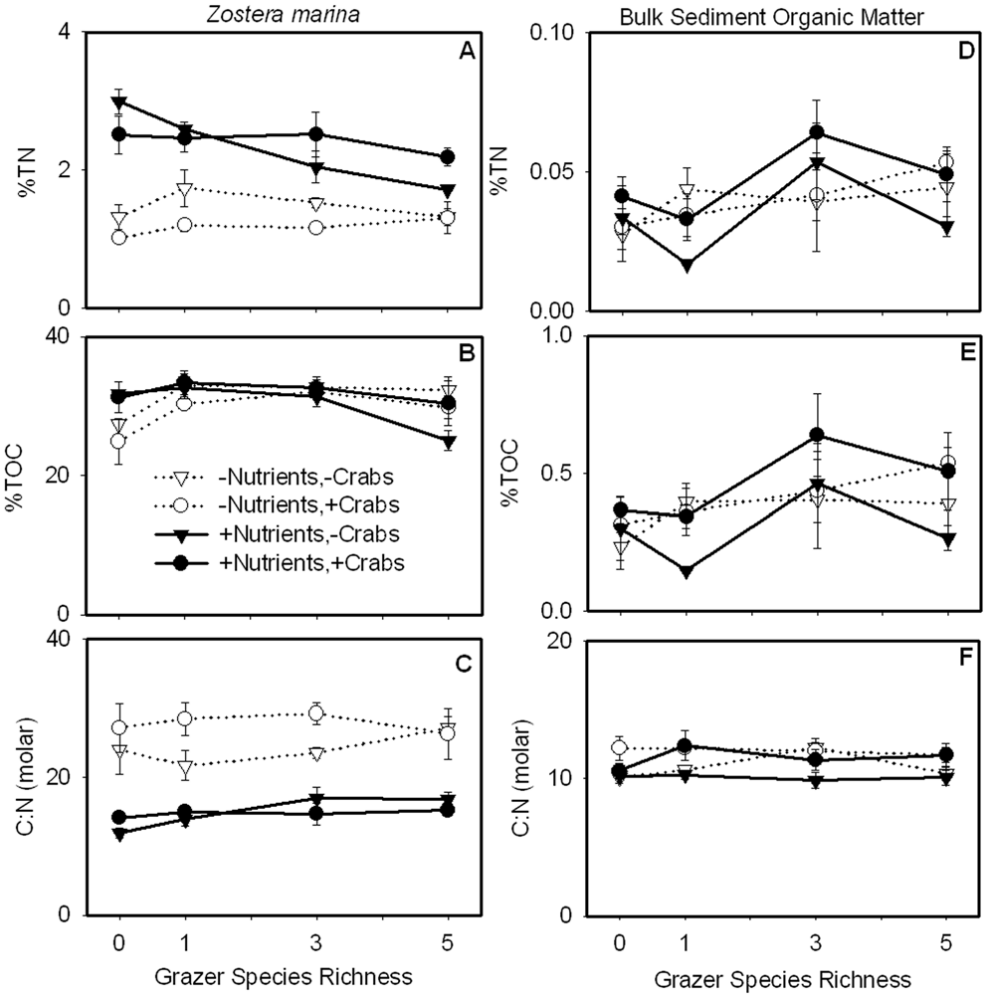
Stoichiometry of *Z. marina* (A–C) and bulk sediment organic matter (SOM; D–F). *Z. marina* %TN (A) was increased by nutrients and decreased by grazers while %TOC (B) was increased by grazers in unenriched treatments. Nutrient additions decreased C∶N (mol∶mol) and, hence, increased the nutritional quality of *Z. marina* (C). Grazer richness influenced SOM %TN (D) and %TOC (E) while crab predators increased %TOC and, consequently, C∶N (F). As SOM had a lower C∶N than *Z. marina* or macroalgae, it is likely that SOM derived from multiple sources of varying quality.

Nutrient enrichment increased the nitrogen content of eelgrass as reflected in higher %TN (ω^2^ = 0.64) and lower C∶N (ω^2^ = 0.71) of *Z. marina* blades (Figs. 3A, C; Table 1). Grazers tended to decrease *Z. marina* %TN in the nutrient-enriched treatments and increased %TOC in the ambient nutrient treatments, resulting in grazer by nutrient interaction effects for both variables (Table S1). Heavy grazing prevented us from obtaining macroalgal samples for nutrient analysis from every tank. However, there was also evidence that nutrient enrichment substantially raised macroalgal quality as the C∶N was 26.92 (±2.02 S.E.; n = 8) in unenriched treatments and 14.77 (±1.17 S.E.; n = 12) in nutrient treatments (data not shown).

Elemental content of SOM was less sensitive than that of primary producers to changes in nutrient levels, predator presence, and grazer richness (Figs. 3D–F; Table 1). Predators generally increased %TOC of bulk SOM (ω^2^ = 0.07), especially in the nutrient-enriched treatments; consequently predators also increased the C∶N of SOM (ω^2^ = 0.25). Grazer richness had an idiosyncratic influence on SOM %TN and %TOC, both being maximized in the three-grazer treatment under nutrient enrichment, whereas grazer richness had no effect on the molar C∶N.

**Figure 4:**
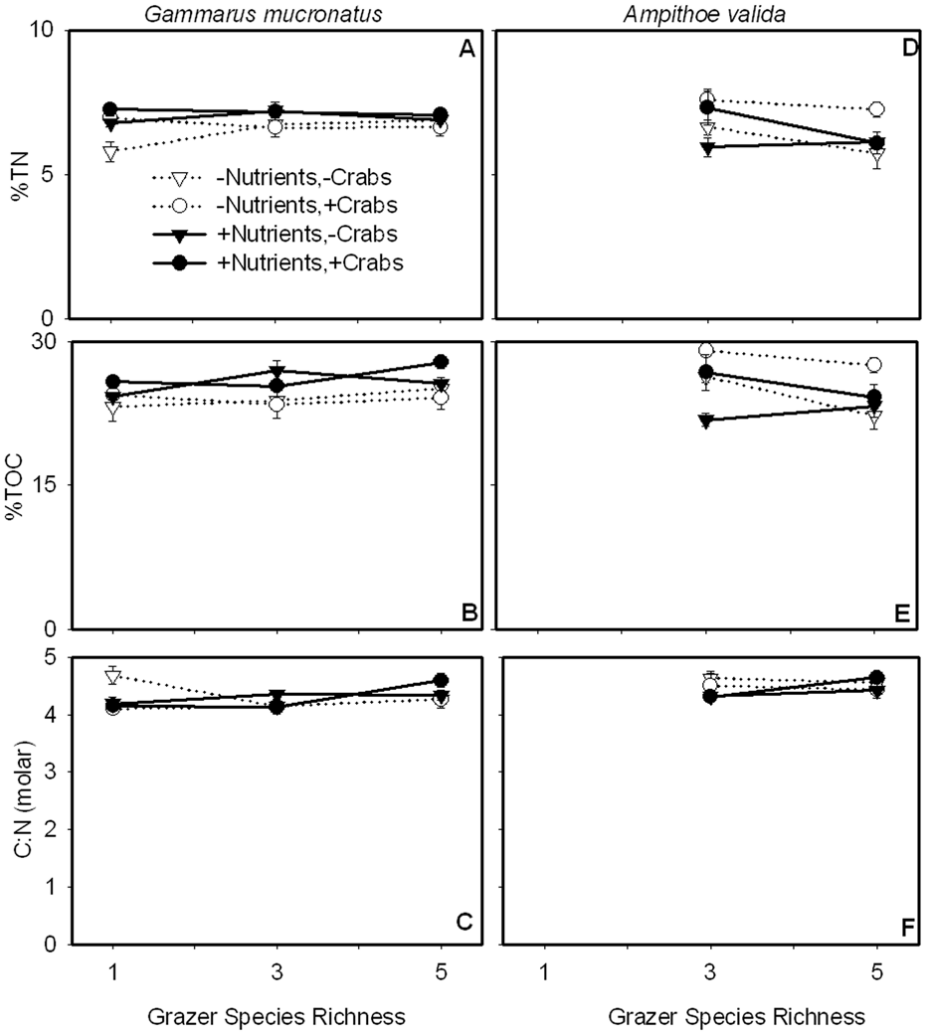
Stoichiometry of *G. mucronatus* and *A. valida*. (A–C) Nutrient enrichment increased *G. mucronatus* %TN and %TOC, but did not affect C∶N. (D–F) Nutrient enrichment decreased and crab presence increased the %TOC of *A. valida* but had no effect on %TN. The C∶N of both grazers was insensitive to nutrient and food web manipulations indicating that grazer stoichiometric ratios were less flexible than primary producers.

At the end of the experiment, the grazer species *G. mucronatus* and *A. valida* differed in their biomass total nitrogen (%TN) and organic carbon (%TOC) content (Fig. 4; Table 1). Fertilizer additions modestly increased %TN (ω^2^ = 0.10) and %TOC (ω^2^ = 0.12) of *G. mucronatus* but reduced the %TOC (ω^2^ = 0.12) content of *A. valida*. Predator presence increased the %TOC content of *A. valida*, perhaps reflecting a shift toward smaller, actively growing amphipod size classes under predation. Despite variations in TOC and TN content, the molar C∶N ratio of grazers was unaffected by any treatment, confirming that the stoichiometric ratio of grazers was more conservative than that of their primary producer food sources.

### Ecosystem fluxes

**Figure 5:**
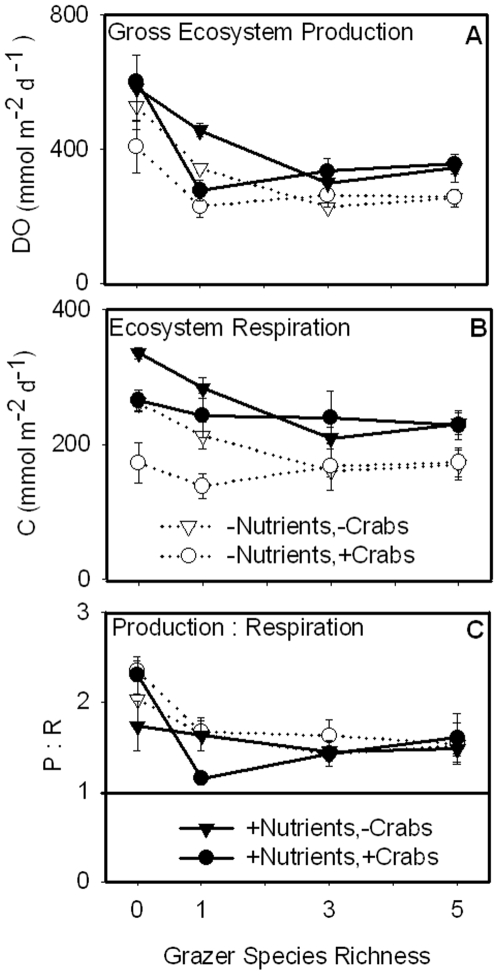
Effects of nutrient enrichment, crab presence, and grazer diversity on ecosystem flux rates. Gross ecosystem production and respiration were increased by nutrient additions and decreased by grazers (A–B); the ratio of production to respiration was also decreased by grazers (C).

Gross ecosystem production (GEP) and respiration were both increased by nutrient enrichment (ω^2^ = 0.11 and 0.34) and reduced by grazers (Figs. 5A, B; Table 1; ω^2^ = 0.53 and 0.14). Importantly, grazer diversity tended to stabilize these ecosystem processes in the face of predation; specifically, crabs decreased GEP (Fig. 5A) and respiration (Fig. 5B) only in the zero- and one- species grazer levels, resulting in a significant predator by grazer interaction for these variables (Table 1). GEP was positively correlated with *Z. marina* and macroalgal biomasses but was unrelated to sediment C∶N (Tables 2, 3). Respiration was positively related to eelgrass abundance and negatively correlated to sediment C∶N. Since respiration was more strongly correlated with sediment C∶N than with net ecosystem production, sediment processes were likely important contributors to ecosystem metabolism in this system (Table 4). GEP was positively correlated to the flux rate of DIN (*p* = 0.011; partial r^2^ = 0.07) but was unrelated to PO_4_^−3^ flux (data not shown). The ratio of production to respiration (P∶R) was generally lower in grazer treatments compared with grazer-free controls (Fig. 5C).

**Table 2:** Regression of daily ecosystem flux rates against biomass of the major primary producer groups.

Ecosystem function	*Z. marina*			Epiphtyic chl *a*			Macroalgae			Benthic chl *a*			Total Model
	Coefficient	r^2^*	*p*	Coefficient	r^2^*	*p*	Coefficient	r^2^*	*p*	Coefficient	r^2^*	*p*	r^2^
GEP	9.34	0.10	**0.001**	4.06	0.00	0.430	9.21	0.27	**<0.001**	34.99	0.01	0.257	0.38
Respiration	3.42	0.06	**0.048**	6.78	0.06	0.054	1.72	0.05	0.092	−4.07	0.00	0.843	0.17
DIN	−2.28	0.07	0.072	3.13	0.03	0.220	0.28	0.00	0.710	8.26	0.01	0.587	0.11
PO_4_^−3^	−0.12	0.06	0.083	0.27	0.08	**0.044**	−0.05	0.03	0.193	−0.16	0.00	0.843	0.18

The coefficient indicates the directionality of the relationship while the partial r^2^ indicates the goodness of fit. Significant p values are in bold. GEP was analyzed as mmol O_2_ m^−2^ d^−1^; respiration as mmol C m^−2^ d^−1^; DIN and PO_4_^−3^ as mmol m^−2^ d^−1^. *Partial r^2^ was calculated by dividing the type III SS by the corrected total SS.

**Table 3:** Regressions of daily ecosystem flux rates against sediment organic matter quality (C∶N; mol∶mol).

Ecosystem function	Sediment C∶N		
	Coefficient	R^2^	*p*
GEP	−29.27	0.08	0.057
Respiration	−21.56	0.20	**0.001**
DIN	−6.61	0.04	0.198
PO_4_^−3^	−0.48	0.08	**0.046**

The coefficient indicates the directionality of the relationship while r^2^ indicates the goodness of fit. GEP was analyzed as mmol O_2_ m^−2^ d^−1^; respiration as mmol C m^−2^ d^−1^; DIN and PO_4_^−3^ as mmol m^−2^ d^−1^.

**Table 4:** Ecosystem respiration as a function of sediment organic matter quality and net ecosystem production (i.e. autochthonous organic matter).

Flux	Sediment C∶N			NEP			Total Model
	Coefficient	r^2^*	*P*	Coefficient	r^2^*	*p*	r^2^
							
Respiration	−20.26	0.17	**0.003**	−33.28	0.06	0.219	0.23

The coefficient indicates the directionality of the relationship while r^2^ indicates the goodness of fit. Significant p values are in bold. NEP and respiration were analyzed as mmol C m^−2^ d^−1^. *Partial r^2^ was calculated by dividing the type III SS by the corrected total SS.

**Figure 6:**
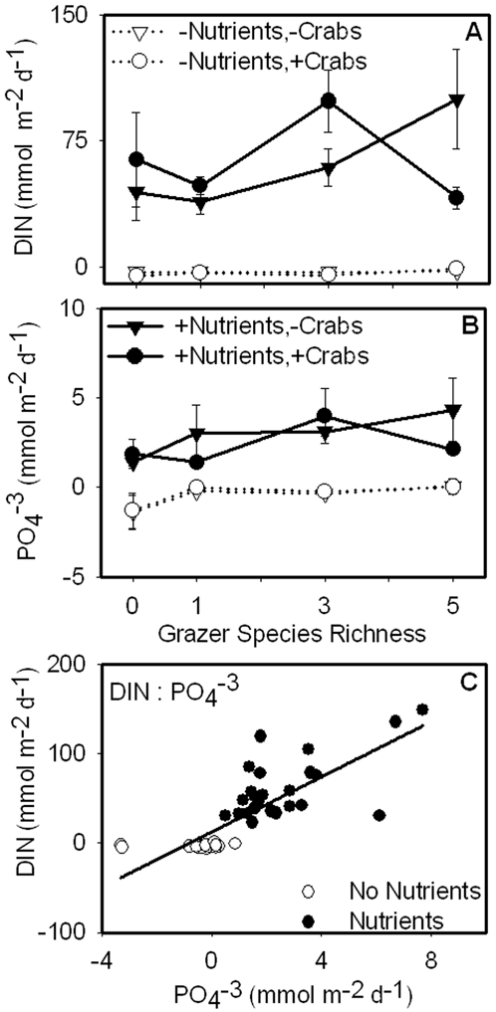
Daily flux rates of DIN and PO_4_^−3^. Nutrient enrichment increased daily flux rates of DIN and PO_4_^−3^ (A–B). DIN and PO_4_^−3^ were positively correlated (r^2^ = 0.64; *p*<0.001; C). The equation of the line was: y = 15.47x + 11.85.

Net DIN and PO_4_^−3^ fluxes increased strongly with nutrient enrichment (ω^2^ = 0.63 and 0.52) but were unaffected by food web manipulations (Figs. 6A, B; Table 1). PO_4_^−3^ flux was positively correlated to epiphyte biomass and negatively related to sediment C∶N while DIN exhibited no patterns in relation to primary producer abundance or sediment quality (Tables 2–3). The slope of the ratio of the fluxes of DIN∶PO_4_^−3^ was 15.47 (r^2^ = 0.64), which was similar to Redfield values.

## Discussion

**Figure 7:**
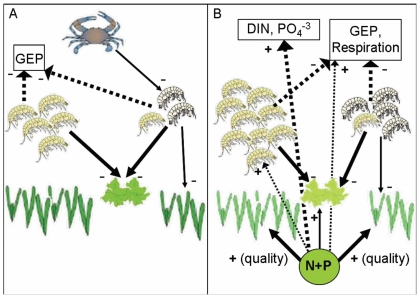
Synthesis of nutrient, crab, and grazer richness effects on major response variables. A and B represent treatments with crab predators and nutrient additions, respectively. Within each panel, the light-colored grazers on the left represent *G. mucronatus* monocultures while the grazers on the right are the multi-species treatments (i.e. minor grazers + *G. mucronatus*). (A) Crab predators reduced minor grazer abundance but had no effect on *G. mucronatus* biomass. Despite the negative effect of crabs on minor grazers, there was no evidence of cascading trophic effects on primary producers or ecosystem process rates. (B) Nutrient additions increased the nutritional quality of primary producers which, likely, indirectly increased *G. mucronatus* biomass. Nutrient amendments increased DIN and PO_4_^−3^ flux rates, respiration, GEP, and macroalgal biomass. In both A and B, gross ecosystem production (GEP) was reduced in all grazer treatments while *Z. marina* biomass was reduced in the mixed grazer species treatments only. Solid arrows are direct effects and broken arrows are indirect effects. Thicker lines represent effects with a ω^2^>0.50; thinner lines represent effects a ω^2^ of <0.50. Low C∶N primary producers are lighter in color than high C∶N algae and *Z. marina.* The − and + symbols indicate the directionality of the effect. See Table 1 for statistical results. *Symbols courtesy of the Integration and Application Network (ian.umces.edu/symbols/), University of Maryland Center for Environmental Science.*

Overall, our experiment showed that nutrient enrichment and grazers each had strong effects on biomass distribution and ecosystem metabolism, whereas predators had relatively weak effects (Fig. 7; Table 1). Importantly, the weak average effects of predation resulted in part from a stabilizing effect of grazer diversity: indirect effects of predatory crabs were smaller in five- vs. one-species grazer treatments for eelgrass biomass (Fig. 1B), gross ecosystem production (Fig. 5A), and ecosystem respiration (Fig. 5B) and each of these inferences is supported by a significant interaction between grazer treatment and predators (Table 1). Since our diversity gradient was designed to simulate a realistic loss of rare species, it involves differences in both species richness and composition, which cannot be separated statistically. Weighed against this, our simulated extinction gradient sidesteps the criticisms repeatedly raised against randomized diversity gradients [see 66] and we believe that it provides a valuable window into potential ecosystem impacts that would result from loss of currently less abundant species. Notably, we found no effect of grazer diversity on biomass accumulation of most primary producers (Fig. 1), in contrast with previous experiments in this system that compared diverse grazer assemblages with the average monoculture [34], [45]; this suggest that the most common grazer (*Gammarus mucronatus*) can compensate for loss of other species in grazing impact. On the other hand, ecosystem productivity and respiration were more stable when the minor grazer species were present (Fig. 5).

Surprisingly, the strongest effect of nutrient enrichment in our experiment was increased accumulation of grazer, not primary producer, biomass (Figs. 1, 2; Table 1). Nutrients evidently moved efficiently through the food chain, increasing the biomass of the grazing amphipod, *G. mucronatus*. Higher grazer biomass likely resulted from nutrient enrichment increasing both primary producer quality (e.g. lower C∶N) and productivity (Figs. 3, 5; Table 1). Grazers, in turn, regulated above-ground algal abundance and this translated into lower rates of ecosystem production and limited response of algal biomass to fertilization (Figs. 1, 5; Table 1). In contrast, fluxes of inorganic nutrients (DIN and PO_4_^−3^) and stoichiometry of eelgrass were strongly influenced by nutrient enrichment and affected little by food web composition (Figs. 3, 6; Table 1). DIN and PO_4_^−3^ were recycled at roughly Redfield proportions (Fig. 6C). Overall, invertebrate grazers strongly affected the productivity and abundance of above-ground primary producers while nutrient enrichment tended to have the strongest influence on the storage and cycling of inorganic nitrogen and phosphorous.

### Effects of nutrient enrichment and food web composition on plant and animal biomass

Grazing was the strongest determinant of above-ground algal biomass. Macroalgal and epiphytic algal biomasses were uniformly low in grazer treatments regardless of nutrient levels (Figs. 1, 7). Grazers also decreased *Z. marina* biomass, but only in the three- and five-species grazer treatments. The negative effect of grazers on algal biomass, and to some extent on *Z. marina* biomass, despite nutrient enrichment, is consistent with previous field experiments [46], [49]. The minor grazers included in the 3- and 5-species treatments, likely reduced *Z. marina* biomass by grazing on leaves. In particular, *Ampithoe valida* is a member of a family known to graze on macroalgae and seagrasses [67], [68]. Since *Z. marina* abundance was similar in the grazer-free controls and in the monocultures of *G. mucronatus*, the latter likely had little effect on eelgrass biomass; this is also consistent with previous experiments [44], [54]. Somewhat unexpectedly, *G. mucronatus* did not significantly increase *Z. marina* biomass by reducing epiphytic algae and, hence, competition for light and nutrients; although there was a trend in that direction under nutrient enrichment (Fig. 1B). Overall, these data corroborate previous studies showing that grazing impacts are strong in seagrass habitats, that grazer species fill different functional roles, and that grazer identity can influence primary producer community composition [68], [69]. More uniquely, our results demonstrate that ecosystem properties are stabilized against top-down perturbations by diverse grazer assemblages (Figs. 1, 5), likely because grazers fill different functional roles [3], [70].

The positive effect of nutrient enrichment on *G. mucronatus* biomass indicated that primary production stimulated by nutrient enrichment was rapidly channeled to grazing invertebrates and, by extension, made available to higher trophic levels. Unlike *G. mucronatus*, minor grazer biomass was not elevated in nutrient enriched treatments (Figs. 2, 7). It is possible that the minor grazers were outcompeted by *G. mucronatus* or that they consumed primary producers that were unresponsive to nutrient additions. Alternatively, changes in grazer abundance may have reflected an early successional sequence and a different pattern might have emerged had the experiment run longer [71].

Blue crab predators had differing effects on individual grazer species, reducing abundances of minor grazers more than that of *G. mucronatus* (Figs. 2, 7). This suggests that the minor grazers were more vulnerable to predation by crabs, perhaps due to their slow population growth rates in the presence of *G. mucronatus*. It is likely that *G. mucronatus* outcompeted the minor grazers for resources, such as food, thereby limiting minor grazer population growth. Despite the negative effect of predators on minor grazer biomass, there was no evidence of a trophic cascade (Figs. 1, 5), probably because the primary producer community reflected the dynamics of the most abundant grazer species, *G. mucronatus*, which was insensitive to predation (Fig. 2B). This finding presents a puzzling contrast with several previous experiments in which crab predators initiated strong trophic cascades, increasing biomasses of macroalgae and sediment microalgae [34], [48], [50]. Although the low vulnerability of *G. mucronatus* to predation may have prevented a trophic cascade, this species was also abundant in the previous experiments that did produce trophic cascades. The absence of predator effects in this experiment underscores the importance of understanding how community composition and different trajectories of species loss affect interactions between successive trophic levels [9], [10], [72].

### Nutrient enrichment and food web composition influence plant and animal stoichiometry

While grazers were the main determinant of primary producer abundance, nutrient enrichment strongly influenced the quality of plant and algal tissues, decreasing the C∶N of *Z. marina* and macroalgae, and likely increasing their nutritional value to grazing invertebrates (Figs. 3, 7). It is unclear whether grazers responded to the higher quality eelgrass leaves (i.e. lower C∶N) since *Z. marina* biomass decreased in both the ambient and nutrient-enriched treatments at higher levels of species richness (Fig. 1). Despite differences in eelgrass leaf quality, the C∶N of grazers did not vary (Fig. 4). This is consistent with previous studies showing that the stoichiometry of invertebrate grazers tends to be less plastic than primary producers [28], [73], [74]. The C∶N ratios of invertebrates are relatively constrained, likely reflecting their body structure and life history [28]. Since the C∶N of grazers was lower than the C∶N of potential food sources (i.e. primary producers and sediments; Figs. 3, 4), it is likely that invertebrate grazers preferentially retained nutrients to maintain an optimal stoichiometric balance.

In addition to bottom-up effects of nutrient enrichment, *Z. marina* carbon and nitrogen content responded to top-down effects of grazers (Figs. 3A, B). For instance, grazers reduced *Z. marina* %TN in nutrient-enriched treatments but increased %TOC in ambient-nutrient treatments (Table S1). Although the mechanism underlying these results is unclear, it is possible that grazers fed selectively on nitrogen-rich tissues leaving nitrogen-deficient tissues behind. Alternatively, grazer damage to eelgrass may have increased the production of carbon-rich secondary metabolites [75], [76], and/or increased carbon-rich structural and/or storage components such as lignin, cellulose (or hemicellulose) and carbohydrates [77].

Changes in the carbon and nitrogen content of eelgrass and macroalgal tissues did not translate into altered SOM quality, as indicated by sediment C∶N values (Fig. 3F). However, sediment C∶N reflects a mixture of contributions from primary producers, animals, microbes, as well as OM present in the sediments at the onset of the experiment.

### Ecosystem metabolism was related to plant biomass and sediment dynamics

A final goal of this experiment was to determine whether ecosystem productivity and fluxes of inorganic nutrients responded to changes in above-ground biomass or in SOM quality. It is important to point out that flux rates measured in this experiment reflected the combination of above-ground processes driven by eelgrass and algae as well as sediment processes. GEP and ecosystem respiration were increased by nutrient additions and decreased by grazers, reflecting similar changes in above-ground primary producer abundance (Figs. 5, 7). Although grazers had a stronger negative effect on GEP than on respiration (Table 1), the experimental system remained net autotrophic as indicated by P∶R values greater than one. Surprisingly, GEP and ecosystem respiration appeared to be influenced by different processes. For instance, GEP was positively related to macroalgal biomass (Table 2) while ecosystem respiration was negatively correlated to surface sediment C∶N (Table 3). Our data are consistent with the hypothesis that sediment processes, rather than above-ground biomass, contribute most to ecosystem respiration (Table 4). However, sediment C∶N (0–1 cm) only explained 20% of the variation in ecosystem respiration. The remainder may be due to microbial processes deeper in the sediments (i.e., below 0–1 cm). Combined, these data indicate that grazers were the strongest determinant of ecosystem production but that nutrient effects on SOM quality likely had indirect effects on respiration rates.

Since patterns of GEP mirrored above-ground plant and algal biomass, we expected daily flux rates of DIN to reflect uptake by plants [78], release by grazers [79], and removal by sediment microbial processes [41]. DIN flux rates were increased by nutrients at all levels of grazer species richness (Figs. 6, 7; Table 1) but, contrary to our hypothesis, were not correlated to the biomass of any of the primary producers or grazers (Table 2; grazer data not shown) nor sediment C∶N (Table 3). In unenriched treatments, DIN fluxes were negative, indicating that inorganic nitrogen was being removed, likely by sediment microbial processes. However, in the presence of nutrient enrichment, DIN fluxes were consistently positive, suggesting high rates of regeneration. It seems likely that active grazing prevented a benthic microalgal response to nutrient enrichment.

In our experimental system, grazers and sediment processes were the most likely contributors to PO_4_^−3^ flux. PO_4_^−3^ is recycled by grazers via waste products and released from sediments under anoxic (or reducing) conditions into the overlying water column [42], [80]. However, the absence of grazer effects on PO_4_^−3^ flux indicated that recycling of PO_4_^−3^ by invertebrate grazers did not contribute much to the daily flux rate (Fig. 6, Table 1). In contrast, PO_4_^−3^ flux was correlated to epiphytic chl *a* and surface sediment C∶N, suggesting that algal biomass and SOM quality were the major influences on this flux (Tables 2, 3). Since epiphytic chl *a* and surface sediment C∶N only cumulatively explain 16% of the variation in PO_4_^−3^ other processes must also be important determinants of PO_4_^−3^ flux. For example, the C∶N composition of surface sediments (0–1 cm) and environmental conditions (e.g., dissolved oxygen concentrations, redox state) were likely not reflective of the entire sediment pool (10 cm depth). If oxygen concentrations decreased with increasing depth, as is typical of coastal sediments, release of PO_4_^−3^ from the deeper anoxic sediments would be likely. Combined, these data suggest SOM quality and likely, sediment reducing conditions, were stronger determinants of daily PO_4_^−3^ flux than grazer richness or predator presence.

In coastal areas, relative fluxes of inorganic nitrogen and phosphorus are generally lower than the Redfield ratio of 16∶1, possibly due to the removal of nitrogen by denitrifying bacteria [42], [80]. In this experiment, daily fluxes of DIN and PO_4_^−3^ were being regenerated at roughly the Redfield ratio, suggesting that algal organic matter was recycled and that denitrification was not important [81]. Overall, our data suggest that both above ground and sediment processes contributed to fluxes of inorganic nutrients and that DIN and PO_4_^−3^ were regenerated in ratios consistent with Redfield organic matter (Fig. 6).

In summary, our results demonstrated that nutrient enrichment and food web composition strongly influenced biomass distribution across trophic levels, stoichiometric ratios of primary producers, and ecosystem metabolism (Fig. 7). Nutrient enrichment increased biomass of macroalgae and epiphytes (chl *a*) which, in turn, increased ecosystem productivity and *G. mucronatus* biomass. Important effects of grazer identity and richness on ecosystem processes are highlighted by intriguing differences between this experiment, which simulated loss of rare species and increasing dominance of *G. mucronatus*, and previous experiments, that used randomly assembled grazer communities. For instance, in this experiment *Z. marina* biomass was lower in the presence of multiple grazer species than with only *G. mucronatus*, whereas in previous experiments using randomized grazer richness gradients eelgrass biomass was unaffected by grazer richness [45], [50]. Duffy et al. [45] compared grazer monocultures and randomly assigned 3-species assemblages to 6-species assemblages and found that grazer biomass and sediment %TOC increased with species richness, on average; these two trends were not observed in the current experiment. Despite differences between experiments in grazer species composition and type of richness gradient, grazers strongly reduced algal biomass in all experiments [45], [50] and reduced DO flux in this and a previous experiment [50]. In this experiment, grazer identity influenced the propagation of nutrient enrichment and predator effects to higher and lower trophic levels, respectively, and grazer diversity tended to buffer several ecosystem properties against such perturbations. This corroborates previous studies demonstrating that grazing can offset the effects of nutrient enrichment on algae [22], [43], [69] and that community composition can markedly affect whether bottom-up controls ascend or top-down controls cascade through a food web.

## Supporting Information

Table S1 Tests of significance and estimated magnitude of effects of nutrient enrichment, food chain length, and grazer species richness and their interactions on biomass, elemental ratios, and daily flux rates.
(0.02 MB PDF)
