# Supplementary material for: Nutrient Enrichment and Food Web Composition Affect Ecosystem Metabolism in an Experimental Seagrass Habitat
Source: PLoS One. 2009 Oct 15;4(10):e7473. doi: 10.1371/journal.pone.0007473 (PMC2759539; doi:10.1371/journal.pone.0007473)
Supplement: Table S1 — Tests of significance and estimated magnitude of effects of nutrient enrichment, food chain length, and grazer species richness and their interactions on biomass, elemental ratios, and daily flux rates. (0.02 MB PDF) [file pone.0007473.s001.pdf]

Table S1. Tests of significance and estimated magnitude of effects (ω^2) of nutrient enrichment, food chain length, and grazer species richness and their interactions on biomass, elemental ratios, and daily flux rates.

Response	Nutrient enrichment			Food chain length			Grazer community			Interactions	Model error	
	p	MS	ω^2	*p*	MS	ω^2	*p*	MS	ω^2		MS	ω^2
Plant biomass												
Zostera marina (AFDM, g)	0.106	25.79	0.02	0.105	25.87	0.02	**<0.001**	182.20	0.49	PxG 0.028 (0.06)	9.30	0.41
Predators	0.260	16.12	0.01				**0.012**	59.31	0.31		11.82	0.62
No predators	0.235	10.11	0.01				**<0.001**	151.75	0.75		6.61	0.26
Macroalgae (AFDM, g)	**0.015**	120.38	0.02	0.458	10.24	0.00	**<0.001**	857.12	0.62	NxG <0.001 (0.13)<br>NxPxG 0.036 (0.03)	18.11	0.21
Nutrients				0.286	26.80	0.00	**<0.001**	918.32	0.83		21.87	0.16
No nutrients				0.849	0.55	0.00	**0.001**	129.57	0.47		14.58	0.47
Epiphytic Chl *a* (μg cm^{-2})	**0.002**	56.36	0.14	0.267	6.63	0.00	**0.004**	27.73	0.19		5.19	0.69
log Benthic Chl *a* (μg cm^{-2})	0.213	0.29	0.01	0.080	0.59	0.05	0.767	0.07	0.00		0.18	1.12
Grazer Biomass												
log Total Grazers (AFDM, mg)	0.189	0.54	0.00	0.371	0.25	0.00	**<0.001**	25.16	0.85		0.30	0.14
Gammarus mucronatus (AFDM, mg)	**<0.001**	3.49E+08	0.18	0.057	4.86E+07	0.02	**<0.001**	2.96E+08	0.45	NxG 0.002 (0.10)	1.23E+07	0.27
Nutrients				0.142	4.74E+07	0.02	**<0.001**	3.13E+08	0.65		1.94E+06	0.34
No nutrients				0.264	8.22E+06	0.01	**0.003**	4.38E+07	0.47		6.10E+06	0.58
log Minor Grazers (AFDM, mg)	0.901	0.00	0.00	**0.002**	4.18	0.47	0.372	0.22	0.00		0.26	0.92
Stoichiometry												
Z. marina %TN	**<0.001**	13.32	0.64	0.232	0.15	0.00	**0.033**	0.33	0.03	NxP 0.041 (0.02)<br>NxG 0.008 (0.05)	0.10	0.23
Nutrients				0.574	0.04	0.00	**0.008**	0.71	0.33		0.13	0.57
No nutrients				**0.014**	0.56	0.21	0.327	0.09	0.02		0.07	0.77
Predators	**<0.001**	9.34	0.79				0.950	0.01	0.00		0.11	0.24
No predators	**<0.001**	4.43	0.48				**0.003**	0.59	0.17	NxG 0.007 (0.13)	0.08	0.22
Z. marina %TOC	0.405	6.65	0.00	0.843	0.37	0.00	**0.012**	40.16	0.15	NxP 0.038 (0.05)<br>NxG 0.011 (0.15)	9.32	0.71
Nutrients				0.203	17.92	0.02	0.053	32.26	0.21		10.15	0.77
No nutrients				0.100	25.93	0.05	**0.008**	48.20	0.37		8.48	0.64
Predators	0.095	42.06	0.08				0.212	22.34	0.07		13.33	0.86
No predators	0.235	8.06	0.01				**0.020**	23.08	0.20	NxG 0.004 (0.32)	5.30	0.47
Z. marina C:N (molar)	**<0.001**	1476.16	0.71	0.088	37.54	0.01	0.399	12.27	0.00		12.10	0.28

SOM %TN	0.852	0.00	0.00	0.144	0.00	0.02	**0.034**	0.00	0.12		0.00	0.86
SOM %TOC	0.918	0.00	0.00	**0.031**	0.15	0.07	**0.037**	0.10	0.12		0.03	0.87
SOM C:N (molar)	0.055	4.64	0.04	**<0.001**	20.34	0.25	0.427	1.11	0.00		1.16	0.72
G. mucronatus %TN	**0.035**	13.04	0.10	0.662	0.51	0.00	0.256	3.78	0.00		2.62	1.05
G. mucronatus %TOC	**0.025**	187.99	0.12	0.855	1.12	0.00	0.136	71.31	0.03		32.89	0.99
G. mucronatus C:N (molar)	0.155	2.11	0.03	0.769	0.09	0.00	0.084	2.71	0.07		0.98	1.05
A. valida %TN	0.421	3.93	0.00	0.766	0.53	0.00	0.958	0.02	0.00	NxPxG 0.045 (0.07)	5.76	1.37
log *Ampithoe valida* %TOC	**0.027**	0.01	0.12	**0.002**	0.02	0.32	0.092	0.00	0.01		0.00	0.65
A. valida C:N (molar)	0.299	0.06	0.01	0.905	0.00	0.00	0.477	0.03	0.00		0.05	1.46
Daily Flux rates												
GEP (mmol O_2 m^{-2} d^{-1})	**<0.001**	1.03E+05	0.11	0.085	1.76E+04	0.01	**<0.001**	1.59E+05	0.53	PxG 0.027 (0.05)	5570.33	0.31
Predators	**0.002**	6.40E+04	0.15				**<0.001**	7.56E+04	0.55		4480.88	0.28
No predators	**0.026**	4.04E+04	0.07				**<0.001**	1.03E+05	0.62		6659.78	0.34
Respiration (mmol C m^{-2} d^{-1})	**<0.001**	6.30E+04	0.34	**0.014**	1.04E+04	0.05	**0.001**	1.01E+04	0.14	PxG 0.012 (0.08)	1542.94	0.42
Predators	**0.001**	3.95E+04	0.44				0.784	843.09	0.00		2352.66	0.67
No predators	**<0.001**	2.44E+04	0.28				**<0.001**	1.59E+04	0.53		733.22	0.21
Production : Respiration (P:R)	0.155	0.19	0.01	0.285	0.11	0.00	**<0.001**	1.05	0.38		0.09	0.58
DIN (mmol m^{-2} d^{-1})	**<0.001**	5.09E+04	0.63	0.906	6.75	0.00	0.201	779.95	0.01	NxPxG 0.041 (0.04)	476.93	0.29
PO_4^{-3} (mmol m^{-2} d^{-1})	**<0.001**	114.04	0.52	0.520	0.87	0.00	0.054	5.85	0.05		2.06	0.46
DIN : PO_4^{-3}	**0.002**	4218.69	0.19	0.403	263.41	0.00	0.506	291.47	0.00		366.53	0.88

When an interaction was significant the dataset was divided according to the interaction (i.e. predators vs. no predators and nutrients vs. no nutrients) and single factor ANOVAs were run
For interactions, P refers to predators, G to grazers, and N to nutrients; ω^2 is listed in parentheses. Significant p values are in bold.
